# Extracorporeal cardiopulmonary resuscitation for in-hospital cardiac arrest: a comparative cohort study from the first ECPR program in Brazil

**DOI:** 10.1016/j.resplu.2026.101386

**Published:** 2026-06-10

**Authors:** Daniel Joelsons, Bruno de Arruda Bravim, Gustavo Saliba, Barbara Rubim Alves, Roberto Rabello Filho, Flávia Baldavira Hirano, Michele Jaures, Renato Carneiro de Freitas Chaves, Rogerio da Hora Passos, ECMO Team HIAE, Ana Laura Jardim Tavares, Barbara Vieira Carneiro, Paula Sepulveda Mesquita, Renan Sandoval de Almeida, Bianca Maria Schneider Pereira Garcia, Caroline Gomes Mol, Cilene Saghabi, Emanuel dos Santos Pereira, Nathalia Lousada Cracel, Raquel Afonso Caserta Eid, Suelen Elaine Uhlig, Barbara Gadioli, Danilo Batista de Almeida, Dhiego Monteiro Diniz, Eliane Silva Maia Fraga, Flavia Veronezi Stankevicius, Gabriela Rodrigues Noronha, Kamila Soares de Almeida, Kelly Cristina da Silva, Kelly Cristina Marques Galvão, Lanara Alves Pereira Leonard, Linaldo Feitoza de Lima, Marcele Liliane Pesavanto, Mariana Fernandes Teixeira Mendes, Matheus Eduardo dos Santos, Patricia Candido da Silva, Raissa Soraya Souza de Oliveira Fernandes, Sabrina Martins Reigota, Sandra Barreto da Silva Costa, Silvia Marinho do Nascimento, Stephanie Peixoto Gracio, Tamara Dresselt Zuccheratto, Tayla de Souza Verceloni Martins, Thais Mirian Dalzogo Benedicto, Walace Souza de Araújo

**Affiliations:** Einstein Hospital Israelita, São Paulo, Brazil

**Keywords:** Extracorporeal cardiopulmonary resuscitation, In-hospital cardiac arrest, Extracorporeal membrane oxygenation, Cardiopulmonary resuscitation, Survival, Brazil

## Abstract

**Background:**

Extracorporeal cardiopulmonary resuscitation (ECPR) is a rescue therapy for refractory in-hospital cardiac arrest (IHCA), but Brazilian data are scarce. We compared ECPR with conventional CPR (cCPR) at the first Brazilian center with a protocolized ECPR program.

**Methods:**

Retrospective single-center cohort at Einstein Hospital Israelita, São Paulo. Adults (18–75 years) with witnessed and refractory IHCA fulfilling institutional ECPR eligibility were included. The cCPR cohort (2017–2025) comprised eligible patients who did not receive ECPR due to absence of 24/7 ECMO coverage; the ECPR cohort (2020–2025) included all protocolized cases. The primary outcome was hospital survival with favorable neurological outcome (Cerebral Performance Category, CPC, of 2 or less). Groups were compared descriptively; survival was estimated by the Kaplan-Meier method with the log-rank test, and the absolute risk reduction and number needed to treat were calculated.

**Results:**

Thirty-eight patients were analyzed (16 ECPR, 22 cCPR). Hospital survival was higher in the ECPR group (43.7% vs 13.6%, *p* = 0.04), corresponding to an absolute risk reduction of 30.1% (95% CI 1.9–58.3) and a number needed to treat of 4. Estimated 30-day survival was 50.0% versus 12.1% (log-rank *p* = 0.02). All survivors achieved CPC of 2 or less. The groups differed substantially at baseline, including in age and sex.

**Conclusions:**

In this first Brazilian single-center cohort, hospital survival was higher and neurological recovery uniformly favorable among patients treated with ECPR for refractory IHCA. These descriptive findings are hypothesis-generating and support the feasibility of a structured in-hospital ECPR program in the Brazilian setting.

## Highlights

• First Brazilian comparative cohort of ECPR versus cCPR in refractory IHCA.
• Hospital survival: 43.7% with ECPR versus 13.6% with cCPR (*p* = 0.04).
• All survivors achieved a favorable neurological outcome (CPC ≤ 2).
• Absolute risk reduction in mortality was 30% with ECPR (NNT of 4).
• ECPR was feasible, with successful cannulation in all 16 attempted cases.

## Introduction

Approximately 300,000 in-hospital cardiac arrests (IHCA) occur annually in the United States, and an estimated 200,000 cardiac arrests occur per year in Brazil.^1^, ^2^ Despite advances in resuscitation science, mortality and neurological disability after cardiac arrest remain unacceptably high, with survival to hospital discharge occurring in only a minority of patients.^3^ Even so, efforts to improve survival and neurological recovery have focused predominantly on out-of-hospital cardiac arrest, leaving IHCA comparatively underexplored.

Extracorporeal membrane oxygenation (ECMO) can temporarily replace cardiac and pulmonary function, maintaining systemic perfusion and oxygenation.^4^, ^5^ Given the poor prognosis associated with prolonged conventional cardiopulmonary resuscitation (CPR), the strategy of implementing veno-arterial ECMO (VA-ECMO) during ongoing cardiopulmonary resuscitation was developed, an approach known as extracorporeal cardiopulmonary resuscitation (ECPR).^6^, ^7^, ^8^ To date, no randomized controlled trials have evaluated ECPR in the IHCA setting. Nevertheless, accumulating evidence from retrospective studies suggests a potentially meaningful benefit, with reported survival rates ranging from 20% to 40%, and a meta-analysis of propensity-matched studies showed that ECPR was associated with reduced in-hospital mortality.^9^, ^10^, ^11^

In Brazil, no center has reported a protocolized ECPR program, and national experience is limited to two case reports.^12^, ^13^ The present study characterizes patients undergoing ECPR for IHCA at a quaternary hospital and compares them with a control group of ECPR-eligible patients treated with conventional CPR (cCPR), according to American Heart Association (AHA) guidelines.^14^ The aim was to evaluate associations between ECPR and hospital survival with favorable neurological outcome.

## Methods

### Study design and setting

We conducted a retrospective, observational analysis at Einstein Hospital Israelita, a quaternary care center located in São Paulo, Brazil. We included all patients who experienced in-hospital cardiac arrest refractory to cardiopulmonary resuscitation performed according to AHA guidelines and who were attended by the hospital's code blue team between 2017 and 2025. This cohort was compared with all patients submitted to ECPR between 2020 and 2025, a period that corresponds to the establishment of the institutional ECMO team in its current configuration. The study was conducted and reported in accordance with the Strengthening the Reporting of Observational Studies in Epidemiology (STROBE) guideline for cohort studies.^15^

### Ethical approval

Ethical approval for this study (Ethical Committee No. CAAE: 91502825.5.0000.0071) was provided by the Ethical Committee of Einstein Hospital Israelita, São Paulo, Brazil (Coordinator: Anna Davison) on 13 February 2026.

### Population

The code blue team is a multidisciplinary team activated for suspected in-hospital cardiorespiratory arrest. A dedicated ECMO team with its current structure and institutional ECPR protocol was established in 2020; therefore, the ECPR cohort comprises cases occurring from 2020 onward, whereas the cCPR cohort was drawn from code blue activations dating back to 2017. ECPR eligibility and activation were governed by a standardized institutional clinical protocol. Allocation to ECPR was therefore determined by predefined clinical criteria in conjunction with operational availability, rather than by individual clinician preference. During the study period, the institution did not provide round-the-clock ECPR coverage; ECPR initiation therefore required, in addition to protocol-defined eligibility, the presence of a qualified cannulating physician on site at the time of the event.

**Fig. 1:**
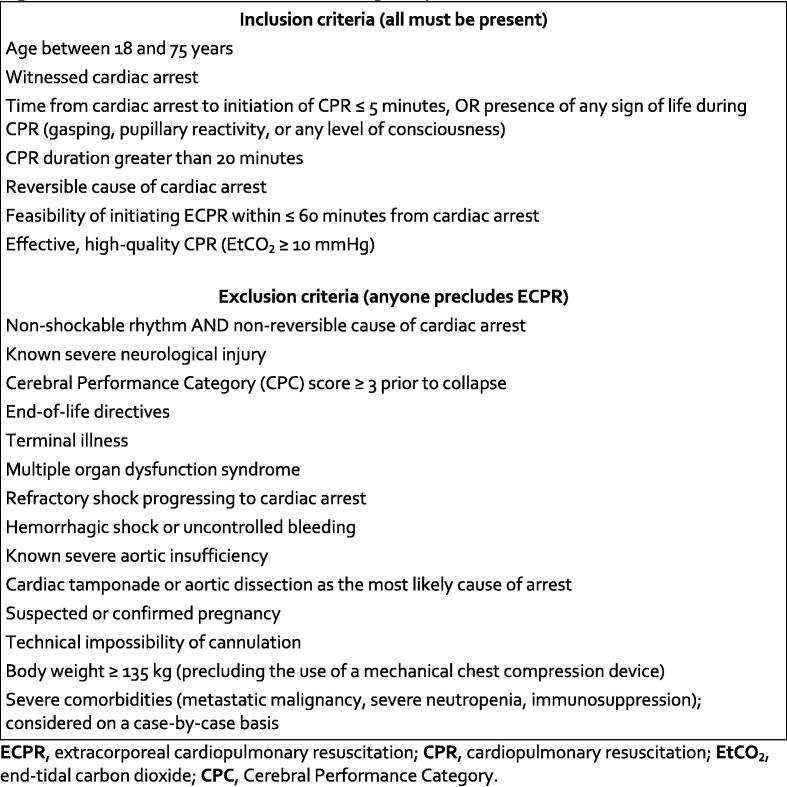
**Institutional criteria for ECPR eligibility**. **ECPR**, extracorporeal cardiopulmonary resuscitation; **CPR**, cardiopulmonary resuscitation; **EtCO**_2_, end-tidal carbon dioxide; **CPC**, cerebral performance category.

Patients were eligible if they experienced IHCA attended by the code blue team and met all institutional ECPR criteria detailed in Fig. 1, including age 18–75 years, witnessed arrest, CPR initiated within 5 min (or any sign of life during CPR), CPR duration greater than 20 min, presumed reversible etiology, and high-quality CPR (EtCO_2_ at least 10 mmHg). Patients were allocated to the ECPR group if they sustained refractory IHCA between 2020 and 2025, fulfilled the above criteria, and had a cannulating physician on site at the time of the event. Patients were allocated to the cCPR group if they experienced IHCA between 2017 and 2025, failed to achieve ROSC after at least 20 min of conventional resuscitation, and fulfilled the same institutional criteria.

Exclusion criteria, detailed in Fig. 1, included a non-shockable rhythm with non-reversible cause, known severe neurological injury, prior CPC ≥ 3, end-of-life directives, terminal illness, MODS, refractory shock, uncontrolled bleeding, technical impossibility of cannulation, and severe comorbidities. In addition, patients who received VA-ECMO for refractory cardiogenic shock after ROSC without ongoing CPR at the time of cannulation were excluded from the ECPR group, and patients in the cCPR group who received VA-ECMO at any point during the hospital stay after ROSC were also excluded.

### VA ECMO implantation and management

When ECPR was indicated and an on-site cannulating physician was available, the ECMO team deployed a Rotaflow centrifugal pump and a Quadrox PLS membrane oxygenator (both Getinge, Germany). A 23 French drainage cannula and a 17 French return cannula were used preferentially. Bifemoral cannulation of the right lower limb was the preferred configuration, although drainage and return could be established through either femoral artery or vein, according to technical considerations or operator judgment. All cannulations were performed percutaneously using the Seldinger technique under ultrasound guidance. Once an ECMO flow greater than 2 L/min was achieved, CPR was discontinued and flow was titrated in conjunction with echocardiographic assessment.

### Data collection and study variables

Variables and data sources were defined a priori. Data were extracted retrospectively from the institutional electronic health records and entered into a standardized REDCap database by the principal investigator alone, to ensure uniform criteria across both cohorts.

For the ECPR group, all VA-ECMO cases registered in the institutional ELSO database between 2020 and 2025 were reviewed, and those corresponding to ECPR were selected for chart abstraction. For the cCPR group, the responsible department provided a list of all code blue activations between 2017 and 2025 involving patients aged 18–75 years with CPR duration greater than 20 min; the corresponding medical records were then reviewed to verify fulfillment of institutional ECPR eligibility criteria. Comorbidities not documented in the medical record were considered absent.

### Outcomes

The primary outcome was survival to hospital discharge with favorable neurological outcome, compared between the ECPR and cCPR groups. Neurological status was assessed using the five-point Cerebral Performance Categories (CPC) scale,^16^ with scores of 1 or 2 classified as favorable and scores of 3 or higher as unfavorable. CPC scores were retrospectively determined from discharge documentation recorded in the electronic medical records. As a secondary outcome, we provided an analytical description of all patients who underwent ECPR, including their clinical profiles, procedural characteristics, and outcomes.

### Statistical analysis

Continuous variables were tested for normality (Shapiro-Wilk). Normal variables are presented as mean ± SD and compared by Student's *t*-test; non-normal variables as median (IQR), compared by Mann-Whitney *U* test. Categorical variables are expressed as absolute frequencies and percentages and were compared using the Pearson chi-square test or Fisher's exact test when expected cell counts were below five. Survival time was defined as the interval from the initiation of CPR to death or hospital discharge, whichever occurred first. Survival up to hospital discharge was estimated using the Kaplan-Meier method, and survival curves were compared between groups using the log-rank test. The absolute risk reduction in hospital mortality and the corresponding number needed to treat (NNT) were calculated with 95% confidence intervals. Missing data (less than 10% per variable) were handled by complete case analysis, with denominators specified in tables. All analyses were performed using Stata version 19.5 (StataCorp, USA).

## Results

### Patient characteristics

Between 2017 and 2025, the code blue team was activated in 198 cases. After applying the eligibility criteria, 38 patients were included in the final analysis: 16 managed with ECPR and 22 with cCPR. The 22 cCPR patients represent ECPR-eligible cases who did not undergo cannulation owing to the absence of an available on-site cannulating physician at the time of the arrest. Among the 16 patients in whom ECPR was initiated, cannulation was successful in all cases, with no cannulation failures.

Baseline characteristics are presented in Table 1. Patients in the ECPR group were younger (median 43.0 years, IQR 32.5–52, versus 67.5 years, IQR 61–70; *p* < 0.001) and more frequently male (93.75% versus 54.55%; *p* = 0.009). The overall comorbidity burden did not differ significantly between groups (81.82% versus 56.25%; *p* = 0.15), although diabetes mellitus was more prevalent in the cCPR group (50% versus 18.75%; *p* = 0.05). Time from admission to cardiac arrest (CA) and the location of the event were comparable between groups, with the catheterization laboratory being the most frequent site of CA in both. Initial rhythm distribution was also similar, with PEA predominating in both groups and shockable rhythms ranking second in the ECPR group. Time from CA to CPR initiation and total CPR duration did not differ significantly between groups. CPR was initiated immediately upon CA recognition in 34 of 36 patients with available data (94.4%), with only two patients experiencing delayed initiation: one in the cCPR group (5 min) and one in the ECPR group (1 min).

**Table 1:** Baseline characteristics of patients undergoing ECPR versus cCPR for in-hospital cardiac arrest.

**Variable**	**cCPR (*n* = 22)**	**ECPR (*n* = 16)**	***p*-value**
**Demographics**			
Age, years	67.5 (61–70)	43.0 (32.5–52)	**<0.001**
Time admission to CA, days	5 (0–10)	1 (0–9.5)	0.83
Male sex	12 (54.55%)	15 (93.75%)	**0.009**
**Comorbidities**			
Any comorbidities	18 (81.82%)	9 (56.25%)	0.15
Diabetes mellitus	11 (50%)	3 (18.75%)	**0.05**
Arterial hypertension	12 (54.55%)	4 (25%)	0.07
Congestive heart failure	2 (9.09%)	3 (18.75%)	0.38
COPD	2 (9.09%)	0 (0%)	0.22
Prior MI	6 (27.27%)	0 (0%)	**0.02**
Prior stroke	1 (4.55%)	0 (0%)	0.39
Dialysis-dependent CKD	2 (9.09%)	0 (0%)	0.22
Pre-cardiac transplant	1 (4.55%)	1 (6.25%)	0.82
Post-cardiac transplant	1 (4.55%)	2 (12.5%)	0.37
Pre-lung transplant	0 (0%)	1 (6.25%)	0.23
Post-lung transplant	0 (0%)	1 (6.25%)	0.23
**Cardiac arrest characteristics**			
Location of CA			0.06
ICU	1 (4.55%)	4 (26.67%)	
Catheterization lab	14 (63.64%)	6 (40%)	
Ward	5 (22.73%)	1 (6.67%)	
Other	2 (9.09%)	4 (26.67%)	
Initial rhythm			0.41
VF/VT	4 (18.18%)	6 (37.5%)	
Asystole	4 (18.18%)	2 (12.5%)	
PEA	14 (63.64%)	8 (50%)	
Suspected cause of CA#			0.09
Cardiac (AMI)	17 (80.95%)	7 (43.75%)	
Pulmonary embolism	2 (9.52%)	3 (18.75%)	
Hypoxemia	1 (4.76%)	1 (6.25%)	
Other	1 (4.76%)	5 (31.25%)	
Total CPR duration, min	44.82 (±18.89)	48.12 (±16.28)	0.58
Immediate CPR initiation after CA (time from CA to CPR = zero min)	20 (95.2%)	14 (93.3%)	1.00
ROSC	5 (25%)	14 (100%)	**<0.001**
**Outcomes**			
Hospital mortality	19 (86.36%)	9 (56.25%)	**0.04**
Neurological outcome (survivors only)			0.49
CPC 1	2 (66.67%)	6 (85.71%)	
CPC 2	1 (33.33%)	1 (14.29%)	
Time from CA to hospital discharge (survivors only), days	21 (13–224)*	26 (22–45)	0.57

Data are presented as median (IQR) for non-parametric continuous variables, mean (±SD) for parametric continuous variables, and *n* (%) for categorical/binary variables.

Non-parametric variables compared using Wilcoxon rank-sum test; parametric variables using independent samples *t*-test; categorical/binary variables using Pearson chi-square or Fisher's exact test.

CA = cardiac arrest; CKD = chronic kidney disease; AKI = acute kidney injury; CPR = cardiopulmonary resuscitation; ROSC = return of spontaneous circulation; ECMO = extracorporeal membrane oxygenation; VF = ventricular fibrillation; VT = ventricular tachycardia; PEA = pulseless electrical activity; AMI = acute myocardial infarction; MI = myocardial infarction; COPD = chronic obstructive pulmonary disease; MODS = multiple organ dysfunction syndrome; CPC = cerebral performance category; IQR = interquartile range; SD = standard deviation.

*cCPR survivors *n* = 3, with individual values of 13, 21 and 224 days.

# Data on suspected cause of CA available for 21/22 cCPR patients.

### Primary outcome

**Fig. 2:**
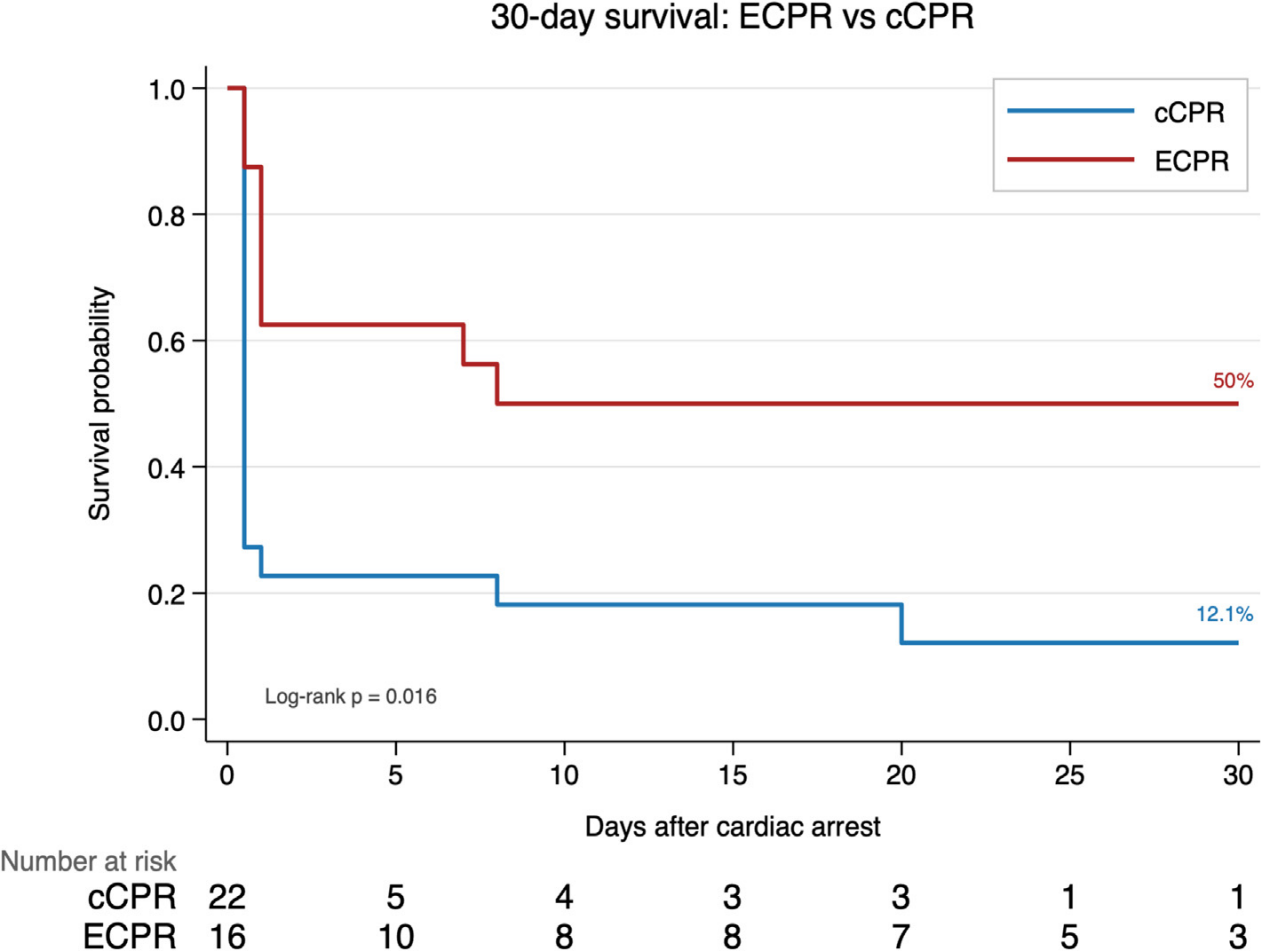
**Kaplan-Meier 30-day survival curves for ECPR versus cCPR following in-hospital cardiac arrest**. Survival probability was estimated using the Kaplan-Meier method and compared between groups using the log-rank test (*p* = 0.02). Numbers at risk are displayed below the *x*-axis. ECPR = extracorporeal cardiopulmonary resuscitation; cCPR = conventional cardiopulmonary resuscitation.

Hospital mortality was lower among patients treated with ECPR than with cCPR (56.3% versus 86.4%; *p* = 0.04). The absolute risk reduction in hospital mortality with ECPR compared to cCPR was 30.1% (95% CI 1.9–58.3), corresponding to an NNT of 4 (95% CI 2–53). Kaplan-Meier survival analysis (Fig. 2) showed higher hospital survival in the ECPR group (log-rank *p* = 0.02), with an estimated 30-day survival probability of 50.0% (95% CI 24.5–71.1) in the ECPR group versus 12.1% (95% CI 2.4–30.2) in the cCPR group. Most deaths occurred within the first 24 h in both groups. Among patients who survived to hospital discharge, all presented a CPC score of 2 or less.

### Secondary outcome

Among ECPR patients, cannulation was initiated by a mean of 21.5 (±13.5) minutes after CA, with a mean cannulation time of 27.4 (±14.0) minutes. The mean of total CPR duration was 48.1 (±16.3) minutes, and median ECMO support duration was 2.5 days (IQR 1–5). Detailed procedural characteristics and complications are presented in Table 2. Within the ECPR group, hospital survival was higher among those with a CPR duration of 40 min or less than among those with longer durations (75% versus 33%; *p* = 0.15), although this difference did not reach statistical significance, likely reflecting the limited size of this cohort (Table 3).

**Table 2:** ECMO procedural characteristics and complications in the ECPR group.

**Variable**	**ECPR group (*n* = 16)**
Time from CA to cannulation start, min	21.54 (±13.54)
Cannulation time, min	27.36 (±14.01)
Total CPR duration, min	48.12 (±16.28)
Venous cannula size, French	23.53 (±2.20)
Arterial cannula size, French	17.47 (±2.10)
ECMO support duration, days	2.5 (1–5)
Reperfusion therapy	5 (35.71%)*
**ECMO complications**	
CNS bleeding	1 (6.25%)
Catheter site bleeding	7 (43.75%)
Air entry into circuit	1 (6.25%)
Seizure	2 (12.50%)

Data are presented as mean (±SD) for parametric continuous variables, median (IQR) for non-parametric continuous variables, and *n* (%) for binary variables.

CA = cardiac arrest; ECMO = extracorporeal membrane oxygenation; ECPR = extracorporeal cardiopulmonary resuscitation; CPR = cardiopulmonary resuscitation; CNS = central nervous system; IQR = interquartile range; SD = standard deviation.

* *n* = 14 with available data.

**Table 3:** Hospital survival by subgroup: ECPR versus cCPR.

**Subgroup**	**cCPR (*n* = 22)**		**ECPR (*n* = 16)**		
	**Survival *n* (%)**	**Death *n* (%)**	**Survival *n* (%)**	**Death *n* (%)**	***p*-value**
All patients	3 (13.6%)	19 (86.4%)	7 (43.8%)	9 (56.3%)	**0.04**
**By initial rhythm**					
VF/VT (shockable)	0 (0.0%)	4 (100%)	4 (66.7%)	2 (33.3%)	**0.03**
PEA	2 (14.3%)	12 (85.7%)	2 (28.6%)	5 (71.4%)	0.43
Asystole	1 (25.0%)	3 (75.0%)	1 (50.0%)	1 (50.0%)	0.54
**By CA location**					
ICU	0 (0.0%)	1 (100%)	2 (50.0%)	2 (50.0%)	0.36
Catheterization lab	1 (7.1%)	13 (92.9%)	3 (50.0%)	3 (50.0%)	**0.03**
Ward	1 (20.0%)	4 (80.0%)	0 (0.0%)	1 (100%)	0.62
Other	1 (50.0%)	1 (50.0%)	2 (50.0%)	2 (50.0%)	1.00
**By CPR duration – ECPR only**					
CPR ≤ 40 min	–	–	3 (75.0%)	1 (25.0%)	0.15
CPR > 40 min	–	–	4 (33.3%)	8 (66.7%)	

All *p*-values by Pearson chi-square test. Between-group comparisons: cCPR vs ECPR within each subgroup. CPR duration analysis restricted to ECPR group (*n* = 16).

CA = cardiac arrest; VF = ventricular fibrillation; VT = ventricular tachycardia; PEA = pulseless electrical activity; CPR = cardiopulmonary resuscitation.

## Discussion

Refractory IHCA lasting more than 20 min is associated with high mortality, and a substantial proportion of survivors present significant neurological sequelae.^17^, ^18^, ^19^ In this context, the 2025 American Heart Association guidelines, the 2025 ILCOR Consensus on Science with Treatment Recommendations, and the 2025 European Resuscitation Council guidelines recognize ECPR as a rescue option for selected patients with refractory cardiac arrest when conventional CPR is failing, in settings where it can be implemented.^14^, ^20^, ^21^, ^22^ In our cohort, hospital survival among patients with refractory CA who underwent ECPR was 43.7% (7/16), compared to 13.6% (3/22) in the cCPR group. However, the groups differed substantially at baseline (notably in age, sex, and comorbidity profile), and allocation depended on protocol eligibility and on-site cannulator availability rather than randomization; these differences preclude causal interpretation. Moreover, all patients who survived to hospital discharge presented a CPC score ≤ 2, indicating that the survival was not achieved at the cost of poor neurological recovery. These results are consistent with outcomes reported by major international centers performing ECPR for IHCA, and in some comparisons even surpass them.^23^, ^24^, ^25^, ^26^

Although this is a retrospective study, no statistically significant difference was observed in the burden of comorbidities between the two groups, despite a higher proportion of diabetic patients and patients with previous myocardial infarction in the cCPR group. The ECPR group, in turn, presented a lower median age, which is in line with the findings of Tona et al.,^27^ who reported a tendency to offer ECMO to younger patients when comparing ECMO with cCPR. Another feature consistent with the literature was that only a small proportion of ECPR cases occurred in the ward setting.^25^

Regarding the initial cardiac rhythm, while shockable rhythms are consistently associated with better outcomes in OHCA scenarios treated with ECPR,^28^, ^29^, ^30^, ^31^ the evidence in IHCA remains controversial, and many centers do not use initial rhythm as a discriminator when deciding whether to proceed with ECPR.^9^, ^23^, ^26^, ^27^, ^32^ This pattern was also observed in our cohort, in which PEA was the most frequent rhythm among ECPR patients, followed by shockable rhythms. A likely explanation is that a substantial share of arrests occurred in the catheterization laboratory during coronary procedures, a setting in which PEA is a common presentation, as well as the possibility of pulmonary embolism as an underlying cause.

The Kaplan-Meier analysis (Fig. 2) reinforces these findings. In both groups, most deaths occurred within the first 24 h after cardiac arrest, reflecting the severity of the initial insult and the high early mortality inherent to refractory IHCA. However, the magnitude of this early drop was substantially smaller in the ECPR group, and the survival curve subsequently plateaued, suggesting that patients who survive the initial post-arrest period with ECMO support have a reasonable chance of sustained recovery. This temporal pattern is consistent with previously described survival trajectories in ECPR cohorts, in which the first hours and days after cannulation represent the critical window for both hemodynamic stabilization and neurological prognostication.

In a post-hoc exploratory analysis among ECPR patients, we observed a trend toward higher hospital survival in those with a CPR duration of 40 min or less compared to those with CPR exceeding 40 min (75% vs. 33%, a 42 percentage-point difference), although this comparison did not reach statistical significance, likely reflecting the limited sample size of the ECPR group (*n* = 16). This magnitude of effect is consistent with prior reports identifying the low-flow interval as one of the strongest modifiable determinants of survival and neurological recovery after refractory cardiac arrest,^33^, ^34^ and aligns with international recommendations suggesting that the benefit of ECPR declines substantially when cannulation is delayed beyond a 40–60 min window.^26^, ^35^, ^36^ Although this subgroup analysis should be regarded as hypothesis-generating rather than confirmatory, given the small number of patients involved, it reinforces the importance of system-level strategies aimed at minimizing low-flow time, such as early activation protocols, dedicated ECPR teams, and 24/7 ECMO availability, all of which are likely to translate directly into improved outcomes.

The observed difference can also be described in terms of the number needed to treat (NNT). In-hospital mortality was 56.3% (9/16) in the ECPR group versus 86.4% (19/22) in the cCPR group, yielding an absolute risk reduction of 30.1% (95% CI 1.9–58.3; *p* = 0.04) and an unadjusted NNT of 4 (95% CI 2–53). In descriptive terms, this corresponds to roughly four patients treated with ECPR rather than cCPR for each additional in-hospital survivor in our cohort. This figure should be read with caution: the wide confidence interval reflects the small sample size, and the non-randomized design with substantial baseline differences between groups precludes a causal reading. Larger studies would be needed to refine this estimate.

This study also contributes to the scarce Brazilian literature on ECPR. National publications have been limited to a fatal emergency department case report,^12^ a systematic review paired with a local cardiac arrest registry,^37^ and a previous case report from our group describing ECPR as a bridge to heart transplantation.^13^ The present cohort represents the first Brazilian case series to directly compare ECPR and cCPR outcomes in refractory IHCA, reflecting the consolidated experience of the only national center with an active in-hospital ECPR program.

This study has several limitations. First, its retrospective, single-center design and modest sample size (*n* = 38) limit statistical power, external validity, and the precision of confidence intervals for the absolute risk reduction and NNT. Second, the inclusion timeframes differed between groups (cCPR 2017–2025; ECPR 2020–2025), and secular changes in post-cardiac arrest care may have influenced outcomes independently of the intervention. Third, retrospective chart abstraction is subject to documentation inconsistencies, particularly for time-sensitive variables. Finally, ROSC in the ECPR group reflects mechanical circulatory restoration rather than spontaneous recovery, limiting direct between-group comparison of ROSC rates. In addition, although the cohort comprised refractory arrests without sustained ROSC after at least 20 min of conventional CPR, the occurrence of transient ROSC during CPR prior to cannulation was not consistently documented in the retrospective records and therefore could not be reliably quantified.

In conclusion, in this descriptive single-center cohort of refractory IHCA, survival to discharge was higher among patients treated with ECPR than with cCPR, and all survivors achieved a favorable neurological outcome (CPC ≤ 2). Given the small sample and the substantial baseline differences between groups, these findings are hypothesis-generating and should be confirmed in larger, multicenter studies. They nonetheless support the feasibility of a structured in-hospital ECPR program in the Brazilian setting.

## CRediT authorship contribution statement

**Daniel Joelsons:** Writing – review & editing, Writing – original draft, Methodology, Investigation, Formal analysis, Data curation, Conceptualization. **Bruno de Arruda Bravim:** Writing – review & editing. **Gustavo Saliba:** Writing – review & editing. **Barbara Rubim Alves:** Writing – review & editing. **Roberto Rabello Filho:** Writing – review & editing, Validation. **Flávia Baldavira Hirano:** Writing – review & editing, Methodology, Data curation. **Michele Jaures:** Writing – review & editing. **Renato Carneiro de Freitas Chaves:** Writing – review & editing, Writing – original draft, Supervision, Methodology, Conceptualization. **Rogerio da Hora Passos:** Writing – review & editing, Writing – original draft, Methodology.

## Funding

This research received no specific grant from any funding agency in the public, commercial, or not-for-profit sectors.

## Declaration of competing interest

The authors declare that they have no known competing financial interests or personal relationships that could have appeared to influence the work reported in this paper.

## Appendix A Members of the ECMO Team HIAE

Ana Laura Jardim Tavares, MD; Barbara Vieira Carneiro, MD; Paula Sepulveda Mesquita, MD; Renan Sandoval de Almeida, MD. Bianca Maria Schneider Pereira Garcia, PT, MSc; Caroline Gomes Mol, PT, PhD; Cilene Saghabi, PT; Emanuel dos Santos Pereira, PT, MCs; Nathalia Lousada Cracel, PT; Raquel Afonso Caserta Eid, PT, PhD; Suelen Elaine Uhlig, PT, MSc; Barbara Gadioli, RN; Danilo Batista de Almeida, RN, MSc; Dhiego Monteiro Diniz, RN; Eliane Silva Maia Fraga, RN; Flavia Veronezi Stankevicius, RN; Gabriela Rodrigues Noronha, RN; Kamila Soares de Almeida, RN; Kelly Cristina da Silva, RN; Kelly Cristina Marques Galvão, RN; Lanara Alves Pereira Leonard, RN; Linaldo Feitoza de Lima, RN; Marcele Liliane Pesavanto, RN; Mariana Fernandes Teixeira Mendes, RN; Matheus Eduardo dos Santos, RN; Patricia Candido da Silva, RN; Raissa Soraya Souza de Oliveira Fernandes, RN; Sabrina Martins Reigota, RN; Sandra Barreto da Silva Costa, RN; Silvia Marinho do Nascimento, RN; Stephanie Peixoto Gracio, RN; Tamara Dresselt Zuccheratto, RN; Tayla de Souza Verceloni Martins, RN; Thais Mirian Dalzogo Benedicto, RN; Walace Souza de Araújo, RN.
